# Two-dimensional finite quantum Hall clusters of electrons with anisotropic features

**DOI:** 10.1038/s41598-022-06093-y

**Published:** 2022-02-11

**Authors:** Orion Ciftja

**Affiliations:** Department of Physics, Prairie View A&M University, Prairie View, TX 77446 USA

**Keywords:** Materials science, Physics

## Abstract

Low-dimensional nano and two-dimensional materials are of great interest to many disciplines and may have a lot of applications in fields such as electronics, optoelectronics, and photonics. One can create quantum Hall phases by applying a strong magnetic field perpendicular to a two-dimensional electron system. One characterizes the nature of the system by looking at magneto-transport data. There have been a few quantum phases seen in past experiments on GaAs/AlGaAs heterostructures that manifest anisotropic magnetoresistance, typically, in high Landau levels. In this work, we model the source of anisotropy as originating from an internal anisotropic interaction between electrons. We use this framework to study the possible anisotropic behavior of finite clusters of electrons at filling factor 1/6 of the lowest Landau level.

## Introduction

Low-dimensional systems in which electrons are restricted to move in less than three spatial dimensions have always attracted great interest as a result of novel theoretical phenomena and potential for technological applications in the field of electronic devices and materials. In particular, a two-dimensional electron gas (2DEG) system where electrons interact with a standard Coulomb interaction potential is one of the most widely studied problems in theoretical condensed matter physics^1,2^. Unexpected behavior occurs when a 2DEG system is subject to a strong perpendicular magnetic field. This is the quantum Hall regime domain where unique magneto-transport features (such as quantization of Hall resistance, etc.) are observed in high mobility samples in strong perpendecular magnetic field at temperatures very close to the absolute zero.

The laws of quantum mechanics explain the emergence of highly degenerate energy levels known as Landau level-s (LL-s) for the case of a charged particle (the electron) undergoing 2D motion in a perpendicular magnetic field. The nature of the quantum state for a system of *N* electrons is, up to certain degree, determined by the so-called filling factor of that state defined as $\nu =N/N_s$ where $N_s$ (which is proportional to the magnetic field) represents the degeneracy of a LL. Values of $\nu =1, 2, \ldots$ (integer) represent integer quantum Hall effect (IQHE) states. The IQHE states are the easiest to explain since their fundamental properties can be described without involving electron–electron interactions. However, further increase of the magnetic field leads to those situations in which $N < N_s$ and filling factor becomes fractional. This is the case where electrons partially occupy only the lowest Landau level (LLL). This is the regime of the fractional quantum Hall effect (FQHE) liquid states which stabilize only due to correlation/interaction effects among electrons^3^.

A 2DEG system at filling factors $0 < \nu \le 1$ can form various quantum phases. The most common quantum phases studied in the literature if one assumes a standard Coulomb interaction potential between electrons are: (1) incompressible liquid composite fermion (CF) states^4–6^ at $\nu =1/3, 2/5, 3/7, \ldots$ and $\nu =1/5, 2/9, \ldots$; (2) compressible Fermi liquid-like states at even-denominator filling factors^7–13^ of the form $\nu =1/2, 1/4$ and 1/6; and (3) Wigner solid states of electrons^14–16^ for $\nu \le 1/7$. Typical electronic liquid states in the LLL do not manifest magneto-transport anisotropy. This means that the experimental obervation of higly anisotropic quantum Hall phases in high LL-s at filling factors $\nu =9/2, 11/2, \ldots$ was quite an unexpected result^17^. It is quite likely that a unidirectional (or striped) charge-density wave (CDW) state^18,19^ stabilizes at these filling factors. It is also plausible that stabilization of an anisotropic electronic liquid crystalline Pomeranchuk-distorted phase^20–22^ gives rise to such an occurrence. This so-called quantum Hall nematic state has been described also as a broken rotational symmetry (BRS) liquid crystalline phase^23–26^.

Standard models for quantum Hall states in the LLL generally consider the electron’s mass (or electron’s effective mass) to be isotropic. By its nature, the Coulomb interaction potential between any two point charges depends only on the separation distance and, thus, is isotropic. However, recent work^27–45^ has articulated the importance of some form of internal anisotropy on the properties of the system. The simplest source of such internal anisotropy can be the presence of an (effective) anisotropic mass^46^ of the electrons. In fact, it can be proven rigorously^47^ that an anisotropic mass of the electrons leads to an effective anisotropic Coulomb interaction potential of a specific form as used in recent quantum Hall studies^48,49^. The objective of the current study is to examine the energetic stability of a liquid crystalline phase that lacks rotational symmetry at filling factor $\nu =1/6$ of the LLL in presence of a degree of anisotropy introduced by an anisotropic Coulomb interaction potential. We choose this particular filling factor since it is very close to the critical filling factor $\nu \approx 1/7$ where a transition to a Winger solid state takes place. We consider small quantum Hall clusters of electrons in a standard standard disk geometry^50,51^. It is assumed that all electrons are spin-polarized.

## Theory and model

Typical liquid states in the LLL such as Laughlin’s states at $\nu =1/3$ and 1/5 as well as Fermi liquid states at $\nu =1/2$, 1/4 and 1/6 are isotropic liquids. They are energetically more stable than any anisotropic counterpart with the understanding that electrons interact via an isotropic Coulomb potential^52,53^. Nonetheless, an anisotropic interaction potential may change the whole picture and stabilize an anisotropic phase that lacks rotational symmetry. Wigner solids, CDW-s or isotropic liquid phases at a transitional regime are expected to be very sensitive to local order. As a result they can be strongly influenced even by weak perturbations. Of this nature is the isotropic Fermi liquid state^54^ at $\nu =1/6$ which is very close to the Wigner solid states that stabilize around filling $\nu =1/6.5$. After all, the energy discrepancies even between fundamentally different quantum Hall phases (for example, CF Fermi liquid versus Bose Laughlin state^55^) are very small. For this reason, we believe that an internal source of anisotropy in the system may have a chance to shift the energy balance to favour the stabilization of an anisotropic phase.

The model under consideration consists of a 2D system of *N* electrons with charge $-e$ $(e>0)$ and mass $m_e$ subject to a strong perpendicular uniform magnetic field so that the filling factor of the LLL is $\nu =1/6$. The electrons are immersed in a uniformly charged disk with area, $\Omega _N=\pi R_N^2$ where $R_N$ is the radius of the disk. The total charge of the disk is positive and equal to $N \, e$ in order to guarantee the overall charge neutrality of the system. The density of the system (number of electrons per unit area) for a given filling factor $\nu$ can be written as:

1 $$\begin{aligned} \rho _0=\frac{N}{\Omega _N}=\frac{\nu }{2 \, \pi \, l_0^2} \ , \end{aligned}$$

where $l_0=\sqrt{\hbar /(e \, B)}$ is the electron’s magnetic length.

The quantum Hamiltonian of the system is written as:

2 $$\begin{aligned} {\hat{H}}={\hat{K}}+{\hat{V}} \ , \end{aligned}$$

where ${\hat{K}}$ is the kinetic energy operator (in a perpendicular magnetic field) and ${\hat{V}}$ is the total potential energy operator. The kinetic energy operator reads:

3 $$\begin{aligned} {\hat{K}}=\frac{1}{2 \, m_e} \, \sum _{i=1}^{N} \left[ \hat{ \vec {p}}_i+e \, \vec {A}(\vec {r}_i) \right] ^2 \ , \end{aligned}$$

where $\hat{\vec {p}}=({\hat{p}}_x, {\hat{p}}_y)$ is a 2D linear momentum operator and $\vec {A}(\vec {r})$ is the vector potential. For a symmetric gauge:

4 $$\begin{aligned} \vec {A}(\vec {r})=\frac{1}{2} ( \vec {B} \times \vec {r} ) \ , \end{aligned}$$

where $\vec {r}=(x,y)$ is a 2D position vector. The magnetic field vector is taken as:

5 $$\begin{aligned} \vec {B}=\left( 0, 0, -B \right) \ , \end{aligned}$$

where *B* is the magnitude of the magnetic field. The choice of the negative sign of $\vec {B}$ allows one to express the polynomial part of the LLL quantum states in terms of the complex variable, $z=x+i \, y$ rather than its complex conjugate where $i=\sqrt{-1}$.

The total potential energy operator is written as:

6 $$\begin{aligned} {\hat{V}}={\hat{V}}_{ee}+{\hat{V}}_{eb}+{\hat{V}}_{bb} \ . \end{aligned}$$

The electron–electron (ee), electron–background (eb) and background–background (bb) interaction potential energy terms are, respectively, written as:

7 $$\begin{aligned} {\hat{V}}_{ee}= & {} \sum _{i<j}^{N} v_{\gamma }(\vec {r}_i-\vec {r}_j) \ , \end{aligned}$$

8 $$\begin{aligned} {\hat{V}}_{eb}= & {} -\rho _0 \, \sum _{i=1}^{N} \int _{\Omega _N} d^2r \, \, v_{C}(\vec {r}_i-\vec {r} ) \ , \end{aligned}$$

and

9 $$\begin{aligned} {\hat{V}}_{bb}=\frac{\rho _0^2}{2} \int _{\Omega _N} d^2r \int _{\Omega _N} d^2r^{\, \prime } \ v_{C}(\vec {r}-\vec {r}^{\, \prime }) \ . \end{aligned}$$

The usual Coulomb interaction potential is denoted as:

10 $$\begin{aligned} v_C(\vec {r}_{i}-\vec {r}_j)=\frac{e^2}{ |\vec {r}_i-\vec {r}_j| } \ , \end{aligned}$$

where $|\vec {r}_i-\vec {r}_j|$ is the separation distance between two point charges and (as customary, the Coulomb’s electric constant is not written). The anisotropic Coulomb interaction potential between electrons has the form:

11 $$\begin{aligned} v_{\gamma }(\vec {r}_{i}-\vec {r}_{j})= v_{\gamma }({x}_{i}-{x}_{j},{y}_{i}-{y}_{j})= \frac{e^2}{\sqrt{\frac{|x_i-x_j|^2}{\gamma ^2}+\gamma ^2 \, |y_i-y_j|^2 }} \ , \end{aligned}$$

where $\gamma$ is a real anisotropy parameter considered to be positive and $(\vec {r}_i-\vec {r}_j)=(x_i-x_j, y_i-y_j)$. Without any loss of generality we consider, $\gamma \ge 1$. The interaction potential above is anisotropic if $\gamma > 1$ and reduces to the isotropic Coulomb potential for $\gamma =1$:

12 $$\begin{aligned} v_{\gamma =1}(\vec {r}_{i}-\vec {r}_{j})=v_{C}(\vec {r}_{i}-\vec {r}_{j}) \ . \end{aligned}$$

In all expressions above, the coordinates $\vec {r}_{i}$ (or $\vec {r}_{j}$) denote the 2D position vectors of electrons while $\vec {r}$ and $\vec {r}^{\, \prime }$ are the 2D disk background coordinates. The position variables of electrons, $\{ \vec {r}_i \}$ extends all over the space while the background coordinates, $\vec {r}$ (or $\vec {r}^{\, \prime }$) are confined within the disk.

We describe the anisotropic state of electrons at filling factor $\nu =1/6$ by means of a phenomenological Fermi liquid wave function with built-in BRS that reads:

13 $$\begin{aligned} \Psi _{\alpha }= & {} \prod _{i>j}^{N} \, (z_i-z_j)^4 (z_i-z_j+\alpha )(z_i-z_j-\alpha ) \nonumber \\&\times \exp {\left( -\sum _{j=1}^{N} \frac{|z_j|^2}{4 \, l_0^2} \right) } det \left| e^{i \, \vec {k}_{n} \, \vec {r}_j} \right| \ , \end{aligned}$$

where *N* is the number of electrons that occupy the *N* lowest-lying plane wave states labeled by the momenta $\{ \vec {k}_{n} \}$ of an ideal 2D spin-polarized Fermi gas, $z_j=x_j+i \, y_j$ is the 2D position coordinate in complex notation and $\alpha$ is, in general, a (complex) parameter that breaks the rotational symmetry of the wave function. This wave function is a generalization of its $\nu =1/2$ counterpart used in an earlier work^23^. It is easy to note that the wave function in Eq. (13) is antisymmetric and translationally invariant, but lacks rotational symmetry (when $\alpha \ne 0$). As a result, such a wave funnction is an obvious starting point to describe a nematic anisotropic liquid state at filling factor $\nu =1/6$ that lacks rotational symmetry.

## Results and discussions

**Figure 1:**
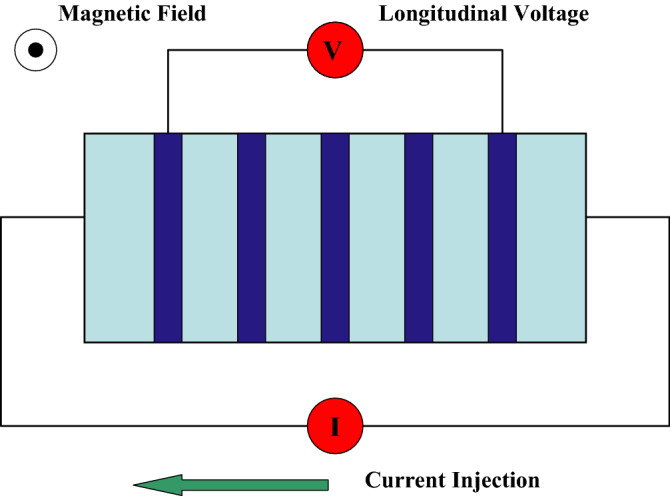
Schematic experimental setup to detect the expected longitudinal magnetoresistance in a 2D quantum Hall sample. The applied magnetic field is perpendicular to the 2D plane of motion of the electron (depicted as stripes) and is directed out of the page (solid dot).

The parameter, $\alpha$ can be considered as a nematic director whose phase is associated with the angle relative to GaAs hard resistance crystalline axis. In our case, we consider $\alpha \ge 0$ to be real so that the system has a stronger modulation in the *x*-direction. Obviously, the values of $\alpha$ that are chosen, for instance, $\alpha =2, 4, \ldots$ are dimensionless expressed in units of magnetic length, $l_0$. If we visualize the electrons as forming layers (or stripes) they will tend to avoid each other along the *x* direction at separation $|x_i-x_j| \approx \alpha$ but aggregate along the *y* direction. A schematic view of the experimental setup for the system under consideration is given in Fig. 1. The anisotropic layering of electrons in this case corresponds to a state with a large magneto-resistance in the direction of the injected current along which response is to be measured.

Our calculations are focused on clusters with a relatevily small number of electrons ranging from $N=5$ to $N=25$. All these systems correspond to filling factor $\nu =1/6$ of the LLL but the number of electrons is suitably chosen to match the closed energy shells of a spin-polarized 2DEG. As explained earlier, the electrons are confined in a uniformly charged background disk and interact with each other via the anisotropic Coulomb potential, $v_{\gamma }(\vec {r}_i-\vec {r}_j)$. Since no analytical results are possible for correlated many-body wave functions of the nature discussed here, we resorted to quantum Monte Carlo (QMC) simulations^56,57^ in disk geometry in order to calculate energy of the BRS liquid crystalline phase described by the BRS wave function in Eq. (13). The energy values obtained this way for a given value of $\gamma \ge 1$ and wave functions with $\alpha > 0$ (BRS anisotropic) are compared to the isotropic liquid counterpart, $\alpha =0$.

The BRS wave function is written in disk geometry but contains a Slater determinant of plane wave orbitals that, strictly speaking, would be appropriate for a square/rectangular geometry in which periodic boundary conditions are applied. To properly select the allowed discrete values of the wave number, $\vec {k}$ in a disk geometry we consider a square with area, $L^2$ equal to the area of the disk where *L* is the length of the square for the specified number *N* of electrons at the given density in such a manner that, $\rho _0=\nu /(2 \, \pi \, l_0^2)=N/L^2$. This way, we determine the values $\vec {k}=(2 \, \pi /L) \, \vec {n}$ where $\vec {k}=(k_x,k_y)$ are the wave numbers and $\vec {n}=(n_x,n_y)$ are the appropriate quantum numbers. The number of electrons, *N* in our calculations is chosen in such a way as to correspond to a complete filled shell in the 2D $\vec {k}$-space for a fully spin-polarized 2DEG system^58^. The QMC calculations, which in this case are variational, allow us to estimate the expectation value of any operator with respect to the given trial wave function. For such a case, $\alpha$ is the variation parameter. The standard Metropolis algorithm is used to calculate the expectation value of, let’s say potential energy, by averaging its value over a large number of configurations of the system. We discard the first 200000 configurations (these are the “thermalization” QMC steps) and then use $2 \times 10^{6}$ configurations for averaging purposes. A QMC step consists of attempts to move one by one all the electrons of the system at random over a certain small pre-determined distance. The acceptance rate of the moves is kept at around $50 \%$. The method of sampling the current wave function is the same as that for Laughlin-like states^56,57^ with the added numerical complexity coming from the Slater determinant that must be re-calculated every time that a particle is moved. The update of the Slater determinant when a particle is moved requires a lot of computer time and, for this reason, we are forced to limit our calculations to relatively small systems.

**Figure 2:**
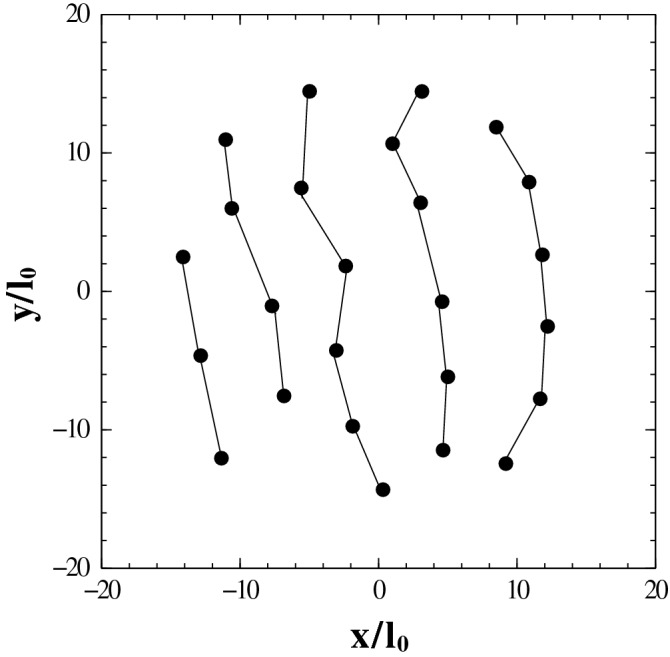
Snapshot of the final configuration of a system of $N=25$ electrons obtained from QMC simulations in a disk geometry. The system corresponds to filling factor $\nu =1/6$ of the LLL and is described by an anisotropic BRS wave function with $\alpha =6$ (in units of the magnetic length, $l_0$). The system manifests signs of layering as shown by the drawn solid lines (that serve as a guide to the eye).

**Figure 3:**
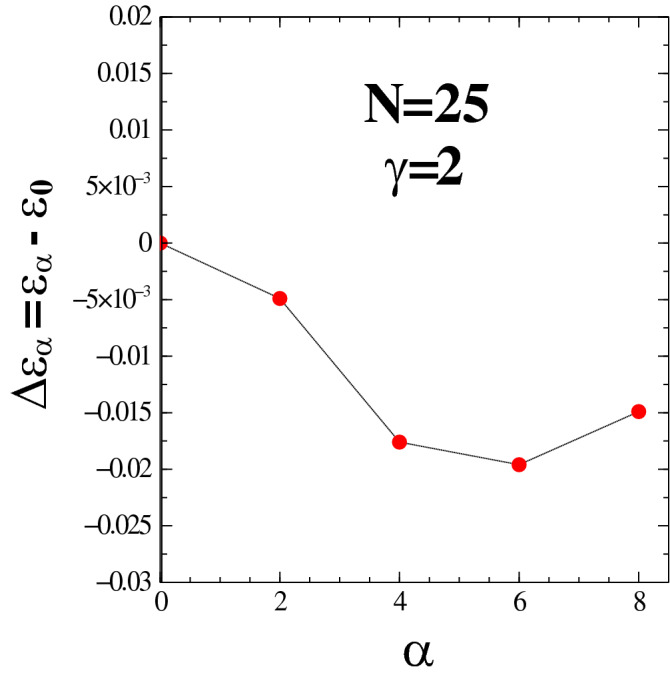
Difference of energy (per electron), $\Delta \epsilon _{\alpha }=\epsilon _{\alpha }-\epsilon _{0}$ as a function of the wave function anisotropy parameter, $\alpha$. The energy, $\epsilon _{\alpha > 0}$ represents an anisotropic BRS liquid state, $\epsilon _{0}$ is its isotropic liquid counterpart. The results correspond to a system of $N=25$ electrons at filling factor $\nu =1/6$ of the LLL. The anisotropic Coulomb potential has an interaction anisotropy parameter, $\gamma =2$. Energies are in units of $e^2/l_0$ The statistical uncertainty of the results is commensurate with the size of the symbols.

Figure 2 provides an attractive visual perspective of how the distribution of electrons at the end of a QMC run mimics the anisotropy of the BRS wave function for the specified value of parameter, $\alpha =6$. Our past experience with QMC simulations of correlated quantum Hall systems has shown that these calculations give very accurate estimate values for quantities of interest including the potential energy (per electron), $\epsilon _{\alpha }=\langle {\hat{V}} \rangle /N$. To this effect, we calculated the energy, $\epsilon _{\alpha }$ for a chosen set of values of the anisotropy parameter, $\alpha$ ranging from 0 (isotropic) to 8 (the largest anisotropic value considered). Based on the results obtained, we calculated the energy difference between the anisotropic BRS liquid crystalline states and their isotropic liquid counterparts:

14 $$\begin{aligned} \Delta \epsilon _{\alpha }=\epsilon _{\alpha }-\epsilon _{0} , \end{aligned}$$

as a function of the anisotropy parameter, $\alpha$.

The results for $\Delta \epsilon _{\alpha }$ corresponding to a system with $N=25$ electrons and $\gamma =2$ are shown in Fig. 3. We estimated that the numerical accuracy of the energy results is up to fifth digit after the decimal point. Being cautious, this value can be used to represent the statistical uncertainty of our results with the size of the symbols drawn in Fig. 3 being commensurate with it. However, we believe that the energy differences are even more accurate than the one reported since the calculation of the difference of two quantities computed separately tends to cancel the respective statistical errors. The results obtained indicate stability of an anisotropic BRS liquid state of electrons for all values of $\alpha$ considered which in this case are $\alpha =0, 2, 4, 6$ and 8.

Note that the magnitude (absolute value) of energy difference, $|\Delta \epsilon _{\alpha }|$ initially increases as $\alpha$ increases from 0 up to the value of 6 (in units of $l_{0}$). However, such energy gain ($|\Delta \epsilon _{\alpha }|$) decreases when $\alpha$ increases from 6 to 8. The parameter $\alpha$ in the BRS wave function is an adjustable parameter that serves as a variational parameter for the energy. For any given value of $\gamma >1$ of the interaction potential we expect the energy to develop a minimum for some optimal value that we denote as $\alpha _0$. As a result the energy for any $\alpha \ne \alpha _0$ will always be larger than that corresponding to the value of $\alpha _0$. This means that, if energy initially decreases as $\alpha$ increases this process does not continue forever. Eventually the energy reaches its minimum at some $\alpha _0$ and then any further increase of the value of $\alpha$ beyond $\alpha _0$ leads to increase of the energy as shown in Fig. 3.

We checked that the same pattern as the one observed for $\gamma =2$ applies to other smaller values of parameter $\gamma$. Specifically speaking, we considered values of $\gamma$ of the form $\gamma =1$ (isotropic Coulomb), $\gamma =1.25$, 1.50 and 1.75. As expected, the wave function with $\alpha =0$ (isotropic Fermi liquid state) has the lowest energy for $\gamma =1$ (isotropic Coulomb potential). However, for all other values, $\gamma =1.25, \ldots$ that were considered in this work, we found out that an anisotropic BRS liquid state has always a lower energy than its isotropic counterpart with optimum value of $\alpha$ close to around 5 or 6 depending on the value chosen for $\gamma$ (note that, because of computational power constraints, we did not try a very accurate optimization of energy as a function of $\alpha$ but we simply choose integer values of $\alpha$ increasing either in steps or 1 or 2 and carried out the necessary QMC simulations).

**Figure 4:**
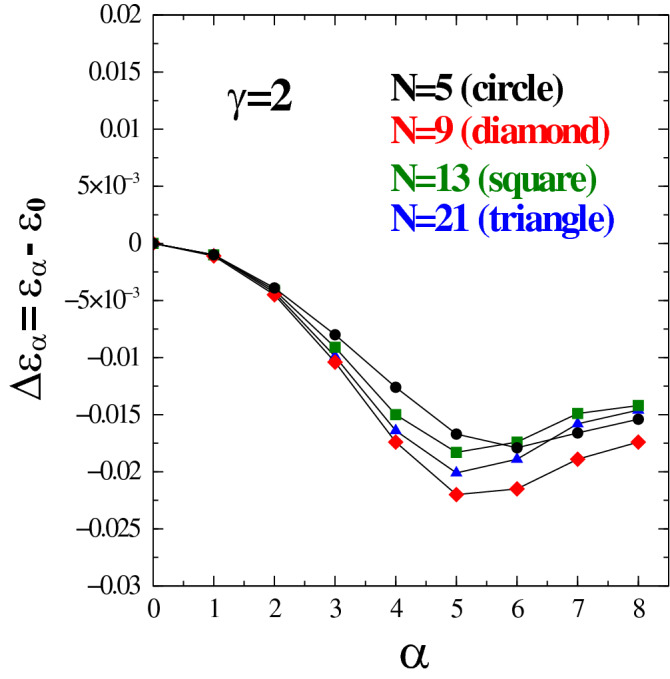
Same as in Fig. 3 but for $N=5$ (black circle), $N=9$ (red diamond), $N=13$ (green square) and $N=21$ (blue triangle).

Similar results were observed for $N=5, 9, 13$ and 21 particles and are shown in Fig. 4. These represent systems that are smaller in size than the $N=25$ case. While the quantitative values of energy differences depend on the size of the system, there are no qualitative differences between various system sizes and various $\gamma$-s. By taking the $\gamma =2$ potential as a representative case, we note that the reduction of energy from $\alpha =0$ (isotropic) to optimal $\alpha$ (BRS anisotropic) is of the order of $10^{-2} \, {e^2}/{l_0}$ for all our systems. Such an energy value is not considered small in the realm of quantum Hall studies. The results obtained indicate that, despite the small size of the systems considered, the energy differences are almost size-independent. This means that there is a good likelihood that the results would also hold in the bulk limit as *N* increases to larger values.

We have seen that, crudely speaking, the value $\alpha _{0} \approx 5$ is the one that optimizes the energy for the $\gamma =2$ case. From simulations with smaller $\gamma$-s, we have seen that $\alpha _0$ decreases when $\gamma$ decreases towards 1 with $\alpha _0=0$ when $\gamma =1$ (isotropic Coulomb potential). The question of what would be the range of $\alpha _0$ does not have an easy answer. However, it is reasonable to expect that the optimal value $\alpha _0$ is determined by the anisotropy ratio of the interaction potential along the “hard” direction relative to the “easy” direction. Assuming $\gamma >1$ and a fixed separation distance, *d* between particles *i* and *j*, then the repulsion is stronger along *x* than *y* direction. In this sense, *x* is the “hard” direction and *y* the “easy” one. It is easy to calculate that:

15 $$\begin{aligned} \frac{\upsilon _{\gamma }(x_i-x_j=d,y_i-y_j=0)}{\upsilon _{\gamma }(x_i-x_j=0,y_i-y_j=d)}=\gamma ^2 \ . \end{aligned}$$

This means that the realistic range of optimal $\alpha _0$ may be expected to be somewhere around $\gamma ^2$. On the other hand, a realistic value of the phenomenological interaction anisotropy parameter, $\gamma$ can be readily provided if the source of internal anisotropy of the 2DEG system is the (effective) mass anisotropy, for instance $m_x$ and $m_y$, along two crystallographic directions. For such a case, we have shown^47^ that $\gamma$ can be directly related to the mass anisotropy ratio via the expression $\gamma ^2=\sqrt{m_x/m_y}$.

Predicting the magnitude of anisotropy, say resistivity/resistance anisotropy, based on our model is not an easy task. This would require an approach similar in spirit to that for half-integer filling factors of the form $\nu \ge 9/2$ that led to an expression of the resistivity ratio, $\rho _{xx}/\rho _{yy}$ which can be directly compared to experimental data^59^. Calculating the hard-to-easy resistivity ratio as a function of the parameters that determine the state (electron density, mobility, effective mass anisotropy, etc.) would require thorough work that we leave for the future given that the approach in Ref.^59^ for the $\nu \ge 9/2$ state cannot be blindly applied to the present $\nu =1/6$ state under consideration.

Formation of clusters of electrons is inherently incorporated into the BRS wave function under consideration. Therefore, it makes perfect sense to study the possibility of anisotropic liquid crystalline phases at any even-denominator-filled state in the LLL in presence of an anisotropic Coulomb interaction potential as that in Eq. (11). However, as argued earlier, the state with filling factor $\nu =1/6$ seems to be the most appealing in this scenario due to its vicinity to the Wigner solid-isotropic liquid phase transition in the LLL. Obviously, the main idea of this study was to give a preliminary glimpse of various possible quantum phases of small systems of electrons for which QMC simulations are feasible in a reasonable amount of time. The simulation time for larger systems of electrons increases very fast given the rather complicated nature of the wave function that incorporates a Slater determinant. Any attempted QMC move of a particle requires an update of the whole column of the determinant that requires a re-calculation. This means that more demanding studies of larger systems of electrons that would require computer time that we do not currently have at disposal will be left to future work.

##  Conclusions

In this study, we focused our attention on small 2D systems of electrons in the quantum Hall regime in which the kinetic energy is practically frozen to the LLL value in absence of interactions. One can create quantum Hall phases of this nature by applying a strong magnetic field in a direction perpendicular to the 2D system at absolute zero temperature. These systems are of great interest to many disciplines and may have a lot of applications in technological fields that involve electrons and structures that operate based on their magneto-transport properties. In fact, the precise nature of the system can be characterized by looking at its magneto-transport response to external probes. By pursuing these lines of discussion, we note that there have been a few quantum phases of electrons seen in experiments on GaAs/AlGaAs heterostructures that manifest anisotropic magneto-resistance with properties that are not fully understood.

We modeled the source of anisotropy as originating from an internal anisotropic interaction between electrons that we call anisotropic Coulomb potential. The degree of anisotropy of the potential is tuned via a phenomenological parameter called $\gamma$ which mimics the effects of this internal degree of anisotropy. The standard isotropic Coulomb potential is recovered for $\gamma =1$. We used this approach to investigate possible anisotropic behavior of finite clusters of electrons in the LLL. In particular, we considered the possibility that the very fragile isotropic Fermi liquid state at filling factor $\nu =1/6$ can be destabilized by an anisotropic Coulomb interaction potential of the form considered. The outcome of this effect would be stabilization of a novel anisotropic quantum phase of electrons with no rotational symmetry. We considered a particular anisotropic liquid crystalline phase with BRS as a good candidate for this scenario. Detailed QMC calculation for smalls system of electrons in a disk geometry support this view for all the values $\gamma > 1$ (anisotropic interaction) and all system sizes considered.

Several studies for quantum Hall systems of electrons in a tilted magnetic field done at filling factor $\nu =9/2$ indicate that the magnitude of the anisotropic perturbation energy introduced in the system is of the order of $10^{-4}$ $e^2/l_0$ which was estimated to be about 10 mK per electron for typical realistic samples and magnetic fields involved in such studies^60,61^. In our investigation of the $\nu =1/6$ state, the typical energy gain of the transition from an isotropic to an anisotropic electron liquid phase is estimated of the order $10^{-2}$ $e^2/l_0$. Assuming that the energy gain would decrease even by an order of magnitude when *N* becomes larger as samples grow toward the bulk size and by accounting the fact that the magnetic length depends on the filling factor, we would still argue that the above energy gain is more or less about 100 mK when converted in thermal scale (multiplied by Boltzmann’s constant, $k_B$). These are ultra-low temperatures, but quite achievable in now-a-days experiments. Energies of such small magnitude are detectable for corresponding low temperatures as shown in recent experiments in quantum Hall samples of electrons at partially filled high LL-s that manifest pronounced anisotropic magneto-resistance^62,63^.

The formalism that leads to the BRS wave function, $\Psi _{\alpha }$ in Eq. (13) for the state with filling factor $\nu =1/6$ can be easily extended to filling factors $\nu =1/8$, 1/10 and so on, by suitably modifying the power of the $(z_i-z_j)$ polynomial factor. It would be interesting to study states like $\nu =1/8$ via a BRS wave function although it is worth noting that, based on our current knowledge, such filling factors are way too deep in the region where Wigner solid phases have a lower energy than the liquid counterparts.

## Data Availability

The data presented in this study are available upon request from the author.
